# Anion Sensing by Solution- and Surface-Assembled Osmium(II) Bipyridyl Rotaxanes

**DOI:** 10.1002/chem.201302886

**Published:** 2013-10-14

**Authors:** Joshua Lehr, Thomas Lang, Octavia A Blackburn, Timothy A Barendt, Stephen Faulkner, Jason J Davis, Paul D Beer

**Affiliations:** [a] Department of Chemistry, University of Oxford South Parks Road, Oxford, OX1 3QZ (UK) E-mail: jason.davis@chem.ox.ac.uk paul.beer@chem.ox.ac.uk

**Keywords:** anion sensing, osmium, rotaxanes, self-assembly, surface immobilization

## Abstract

We report the preparation of [2]rotaxanes containing an electrochemically and optically active osmium(II) bipyridyl macrocyclic component mechanically bonded with cationic pyridinium axles. Such interlocked host systems are demonstrated to recognise and sense anionic guest species as shown by ^1^H NMR, luminescence and electrochemical studies. The rotaxanes can be surface assembled on to gold electrodes through anion templation under click copper(I)-catalysed Huisgen cycloaddition conditions to form rotaxane molecular films, which, after template removal, respond electrochemically and selectively to chloride.

## Introduction

Stimulated by the promise of mechanically interlocked molecular architectures with potential employment in future nanotechnological applications such as in the development of molecular machines and switches, the interest being shown in their construction is ever increasing.[1] Rotaxane and catenane species can also, however, be designed to function as selective host systems whereby the topologically unique interlocked three-dimensional cavities are exploited to selectively recognise specific guest species.[2] In previous work, we have utilised anion templation to construct a range of [2]rotaxanes and [2]catenanes, which, upon removal of the template, bind anions strongly and selectively in competitive solvent mixtures.[3] Furthermore, selective anion binding can be exploited to bring about triggered motion within the interlocked supramolecular architectures, underlining the possible application of these systems in the preparation of molecular switches.[4] To sense and monitor anion binding (and/or binding-triggered motion), it is desirable to integrate a redox- or photoactive reporter group in proximity to the interlocked anion-binding site. Examples of interlocked hosts with the capability of sensing anions by electrochemical[5] or optical[3b, 4, 6] methods are rare.

**Figure 1:**
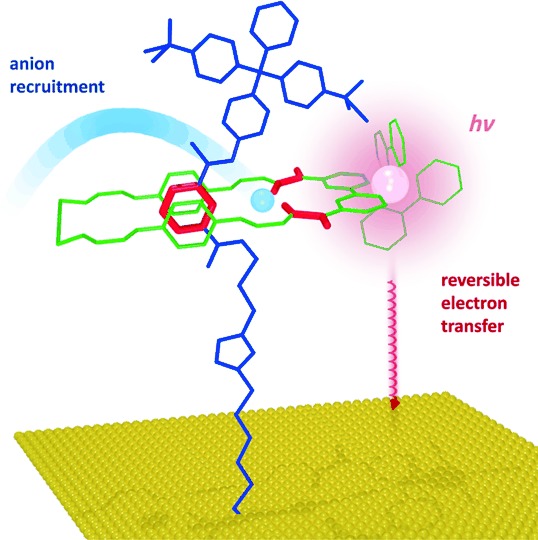
Schematic representation of the recognition and sensing of anions for a surface-bound rotaxane. Axle in blue, macrocycle in green, with anion binding functionalities shown in red.

Herein, we report the preparation of the first anion-templated [2]rotaxane incorporating an optically- *and* electro-active osmium(II) tris(bipyridine) reporter group. After removal of the anion template, selective anion binding is investigated by monitoring the optical and electronic output from the osmium(II) tris(bipyridine) reporter moiety. To develop and fabricate a prototype molecular sensory system, it is highly desirable to effectively immobilise the interlocked host on a substrate. Surface association removes complications associated with Brownian motion, typically increases conformational rigidity, and will underpin potential applications of such molecular architectures and, ultimately, device integration.[1b] To date, only a limited number of surface-bound interlocked structures have been reported.[5b, c, 7] Hence, we additionally report the anion-templated assembly of these electrochemically active osmium(II) bipyridyl rotaxane structures on gold substrates and, after template removal, the specific reporting of anion recruitment from solution (Figure 1).

## Results and Discussion

**Design of the system**: The target design for the mechanically bonded host features the incorporation of the osmium(II) tris(bipyridine) reporter group into the macrocyclic component of a rotaxane molecular framework containing a convergent hydrogen-bond-donor anion-binding interlocked cavity. The macrocycle contains the 4,4′-bis(amide)-2,2′-bipyridyl motif for coordination to the osmium(II) metal centre and electron-rich hydroquinone units to facilitate supplementary secondary aromatic donor–acceptor interactions with the electron-deficient positively charged pyridinium axle.[3a] The choice of the osmium(II) bipyridyl reporter group comes from its established electro- and photochemical properties, making the system an attractive probe to sense the anion-binding event.

**Scheme 1:**
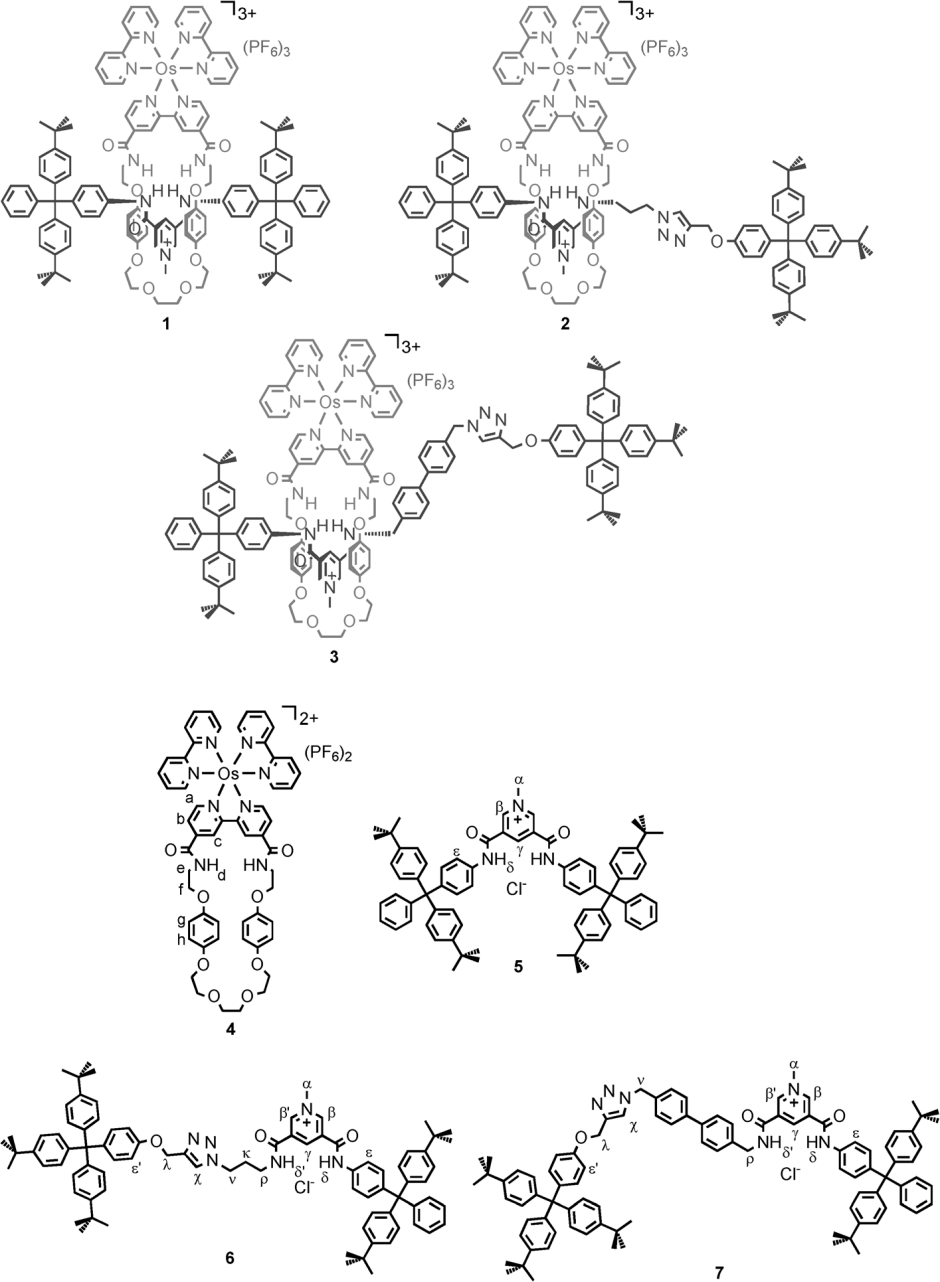
The rotaxanes, macrocycle and axles studied.

**Synthesis of the [2]rotaxanes**: Three distinct osmium(II) bipyridyl (bipy) [2]rotaxanes, **1**–**3**, were prepared by clipping and stoppering anion-templated synthetic methodologies, comprising the same macrocycle component and different axle lengths (Scheme 1).

**Scheme 2:**
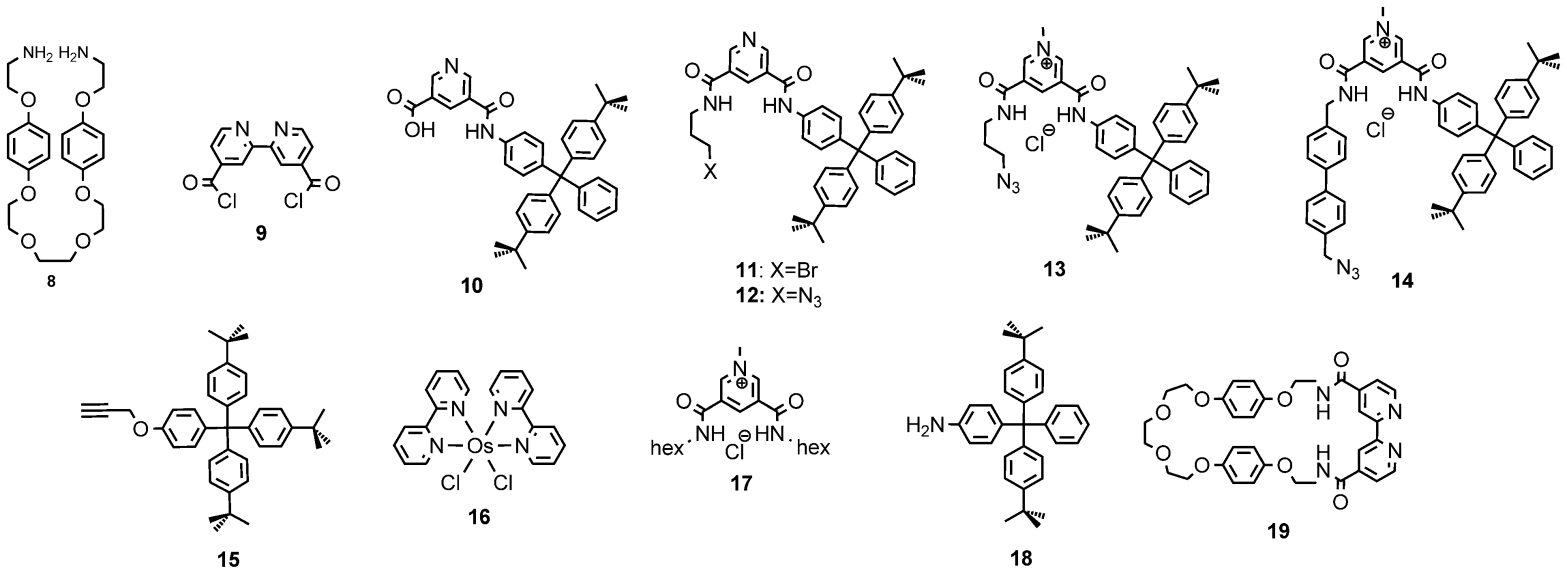
Synthetic precursors 8–19.

Rotaxane **1** was obtained in two steps by a chloride-anion-templated “clipping” reaction between axle[3a] components bis(amine) **8** and 4,4′-bis(chlorocarbonyl)-2,2′-bipyridine **9** in the presence of Et_3_N in dry dichloromethane (Scheme 2 and Scheme S1 in the Supporting information). The crude metal-free rotaxane intermediate was then treated with [Os(bipy)_2_Cl_2_], **16**, in an EtOH/H_2_O mixture at reflux to give, after anion exchange using 0.1 m aqueous NH_4_PF_6_, rotaxane **1** in 6 % overall yield. Macrocycle **19** was synthesised in 37 % yield by condensation of the bis(amine) **8** with bis(acid chloride) **9** in the presence of Et_3_N and template **17** in dry dichloromethane. Reaction of macrocycle **19** with **16** in an EtOH/H_2_O mixture at reflux afforded, after anion exchange, macrocycle **4** in 74 % yield. The reaction of carboxy–terphenyl amide pyridine derivative **10**[3f] with oxalyl chloride produced the corresponding acid chloride, which upon condensation with 3-bromopropylamine hydrobromide in the presence of Et_3_N in dry dichloromethane gave bis(amide) **11**. This was converted to its azide derivative **12** by reaction with sodium azide in dimethylformamide. Reaction of compound **12** with methyl iodide at reflux, followed by anion exchange using 0.1 m aqueous NH_4_PF_6_, gave the desired axle precursor **13**. An analogous condensation reaction between the acyl chloride derivative of **10** and 4′-(azidomethyl)biphenyl-4-yl-methanamine, followed by methylation and anion exchange, produced axle precursor **14** (Scheme 2).

**Figure 2:**
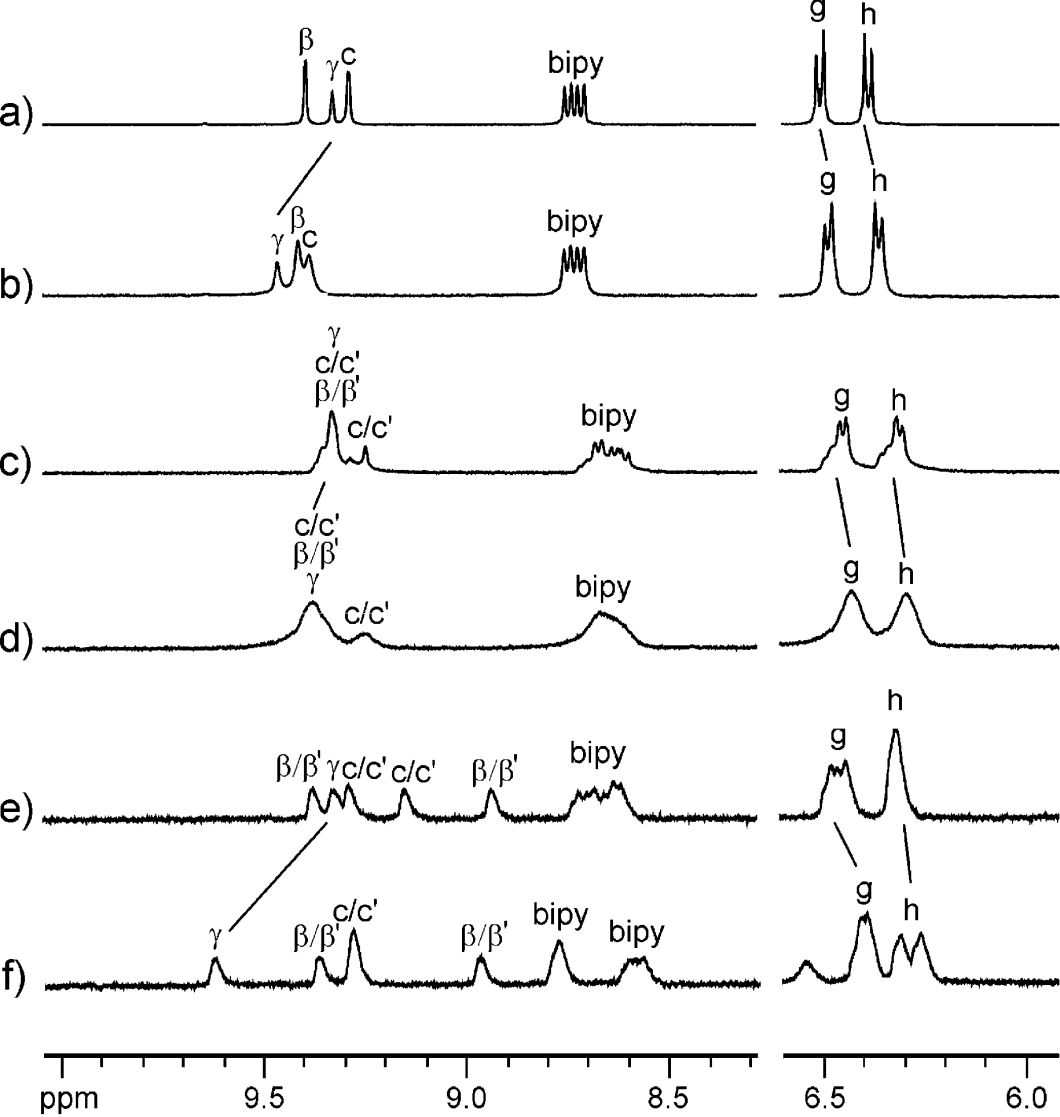
^1^H NMR spectra (500 MHz, [D_6_]acetone/D_2_O (7:3), 293 K) of [2]rotaxanes 1 (a), 2 (c), 3 (e) and their chloride complexes 1 Cl (b), 2 Cl (d), and 3 Cl (f) after addition of one equivalent of chloride.

Rotaxanes **2** and **3** were obtained in 16 and 72 % yield, respectively, by “mono stoppering” reactions followed by washing with NH_4_PF_6_/H_2_O to remove the chloride template (see the Supporting Information, Scheme S1). Axle precursors **13** and **14** were added to macrocycle **4** leading to chloride-anion-templated pseudo-rotaxane assembly. Subsequent copper(I)-catalysed azide–alkyne cycloaddition (CuAAC) reactions in dry dichloromethane using alkyne stopper **15**, copper(I) tetrakis(acetonitrile) hexafluorophosphate, tris(benzyltriazolylmethyl)amine (TBTA) and diisopropylethylamine afforded the desired rotaxanes **2** and **3**. Rotaxanes **1**–**3** were characterised by NMR spectroscopy (^1^H, ^13^C, ^19^F and ^31^P) and by mass spectrometry (MALDI-TOF). ^1^H NMR spectra of rotaxanes **1**–**3** (Figure 2) reveal splitting and an upfield shift of hydroquinone protons of the macrocycle component, owing to aromatic donor–acceptor interactions between the electron-rich hydroquinone groups and the electron-deficient pyridinium moiety of the axle, characteristic of an interlocked structure.

**Anion-binding studies in solution**: Anion binding was probed by using NMR, luminescence and electrochemical methods in assorted solvents as dictated by various factors. These techniques all have dramatically different limits of detection and different requirements from the system. In the case of NMR titration, initial assessments in acetonitrile revealed strong association with anions, compounded by the low solubility of the generated complexes, a fact that necessitated the use of a more competitive aqueous solvent mixture, [D_6_]acetone/D_2_O (7:3).

Luminescence spectroscopy, offering much lower detection limits, presents a quantum yield weighted average; as a consequence, the response of the observed luminescence to anion concentrations varies between solvents. This is particularly true in the case of osmium tris(bipy) complexes, in which solvation of the excited state plays an important role in determining the form of the spectrum.[8] Small quantities of water were added to the acetonitrile mixtures; it was found that the addition of 3 % water produced optimal and reproducible changes to the osmium emission spectrum.

In the case of electrochemistry, the need for a large potential window to resolve bipy-ligand-based voltammetry and the use of relatively concentrated electrolytes dictated the use of acetonitrile as a solvent system.

The results of these studies are discussed in detail below.

**^1^H NMR investigations**: Preliminary ^1^H NMR anion titration experiments between rotaxanes **1**, **2** and **3** and various anions (Cl^−^, AcO^−^ and H_2_PO_4_^−^) were performed in competitive [D_6_]acetone/D_2_O (7:3) solvent mixtures. The addition of one equivalent of tetrabutylammonium (TBA) chloride resulted in a downfield shift of inner protons γ (0.13, 0.05 and 0.29 ppm) and c (0.10, 0.05 and 0.34 ppm) for rotaxanes **1**, **2** and **3**, respectively, indicative of halide binding within the rotaxane’s interlocked binding cavity (Figure 2).

**Figure 3:**
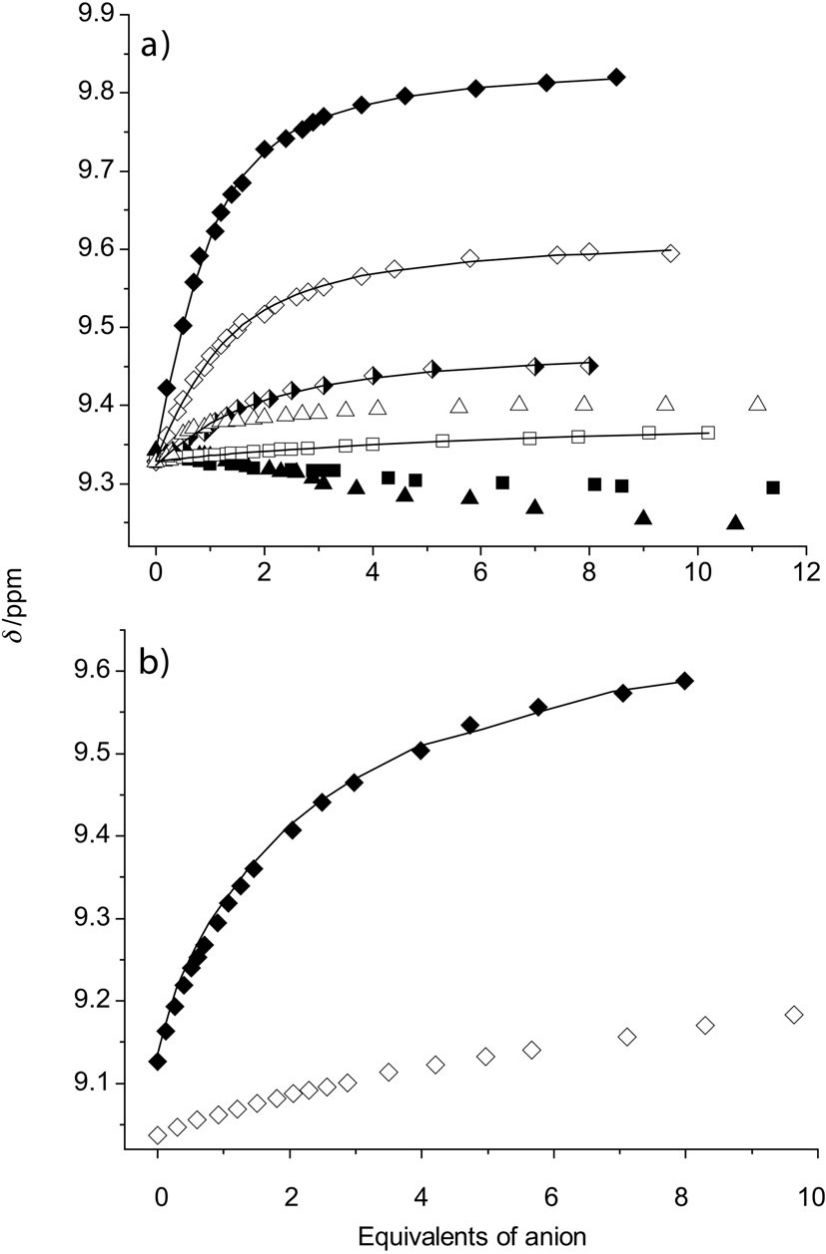
a) Chemical-shift perturbation of proton γ upon addition of Cl^−^ (⧫), AcO^−^ (▪) and H_2_PO_4_^−^ (▴) (as the TBA salt) to a solution of [2] rotaxane 1 (unfilled), 2 (half-filled) and 3 (filled) in 7:3 [D_6_]acetone/D_2_O at 293 K. b) Chemical-shift perturbation of proton c upon addition of Cl^−^ (as the TBA salt) to a solution of macrocycle 4 in 7:3 [D_6_]acetone/D_2_O (◊) and in 9:1 [D_6_]acetone/D_2_O (⧫) at 293 K. Symbols represent experimental data; continuous lines represent calculated curves.

In addition, modest upfield perturbations of the macrocyclic hydroquinone protons of the respective rotaxane were observed, g (−0.02, −0.02 and −0.06 ppm) and h (−0.03, −0.02 and −0.03 ppm) for **1**, **2** and **3**, respectively. When oxoanions such as acetate or dihydrogen phosphate where added, only a small perturbation was observed for inner protons γ. In the case of rotaxane **3**, the γ protons move upfield, an indication of the oxoanion binding on the periphery of the rotaxane.[9] WinEQNMR2 analysis[10] of the binding isotherms with chloride and acetate anions, obtained by monitoring the chemical-shift perturbation of proton γ of the axle component of the respective rotaxane versus equivalent of anion (Figure 3), determined 1:1 stoichiometric association constants (Table 1). It proved impossible to obtain quantitative binding data from any of the dihydrogen phosphate titration experiments, indicative of a complex equilibrium binding process (chemical shifts observed were too small, association constants <100 m^−1^, to be sensibly fitted within error).

**Table 1:** Association constants [m^−1^] of [2]rotaxanes 1, 2 and 3 in 7:3 acetone/D_2_O and macrocycle 4 in 9:1 acetone/D_2_O with Cl and AcO (as TBA salts) at 293 K^[a]^

	Cl^−^	AcO^−^
**1**	750	50^[a]^
**2**	250	–
**3**	1400	–^[d]^
**4**^[b]^	210^[c]^	–^[d]^

[a] Obtained by monitoring proton γ. Error <15 % (for all values). [b] Determined by monitoring proton c. Error <10 %. [c] In 9:1 acetone/D_2_O. [d] Chemical shift changes were too small to be reliably fitted.

The results indicate selectivity towards Cl^−^, with only weak and peripheral association with AcO^−^ and H_2_PO_4_^−^. The oxoanions are too large to penetrate the interlocked binding domain, whereas chloride anions are of complementary size and shape leading to strong binding even in this competitive 30 % aqueous solvent mixture.

Comparing the strength of chloride anion binding for the three rotaxanes, the interlocked host **3**, containing a rigid biphenyl axle linkage, exhibits significantly stronger binding. This may reflect the optimal degree of preorganisation of the interlocked binding domain of the respective rotaxane as determined by the nature of the axle component. The relatively more preorganised binding sites of rotaxanes **1** and **3**, as compared with the flexible propyl-linked axle component present in **2**, potentially enhances chloride binding. Steric congestion could be responsible for **1** being a relatively inferior halide-complexing reagent in comparison with **3**.

The binding of chloride anions by macrocycle **4** was also investigated (Table 1 and Figure 3 b). As expected, the binding is very weak in 7:3 acetone/water, as shown by the small downfield shift of protons c, 0.03 ppm after addition of one equivalent of chloride. The ^1^H NMR titration experiment was repeated in 9:1 acetone/water, a less competitive solvent mixture, which enabled a 1:1 stoichiometric association constant of 210 m^−1^ to be determined. Analogous titrations with acetate anions revealed only small perturbations of the macrocycle proton, indicative of weak binding. These observations serve to highlight the rotaxanes’ potency for chloride anion recognition as a consequence of their three-dimensional binding cavities being capable of encapsulation of this guest.

**Figure 4:**
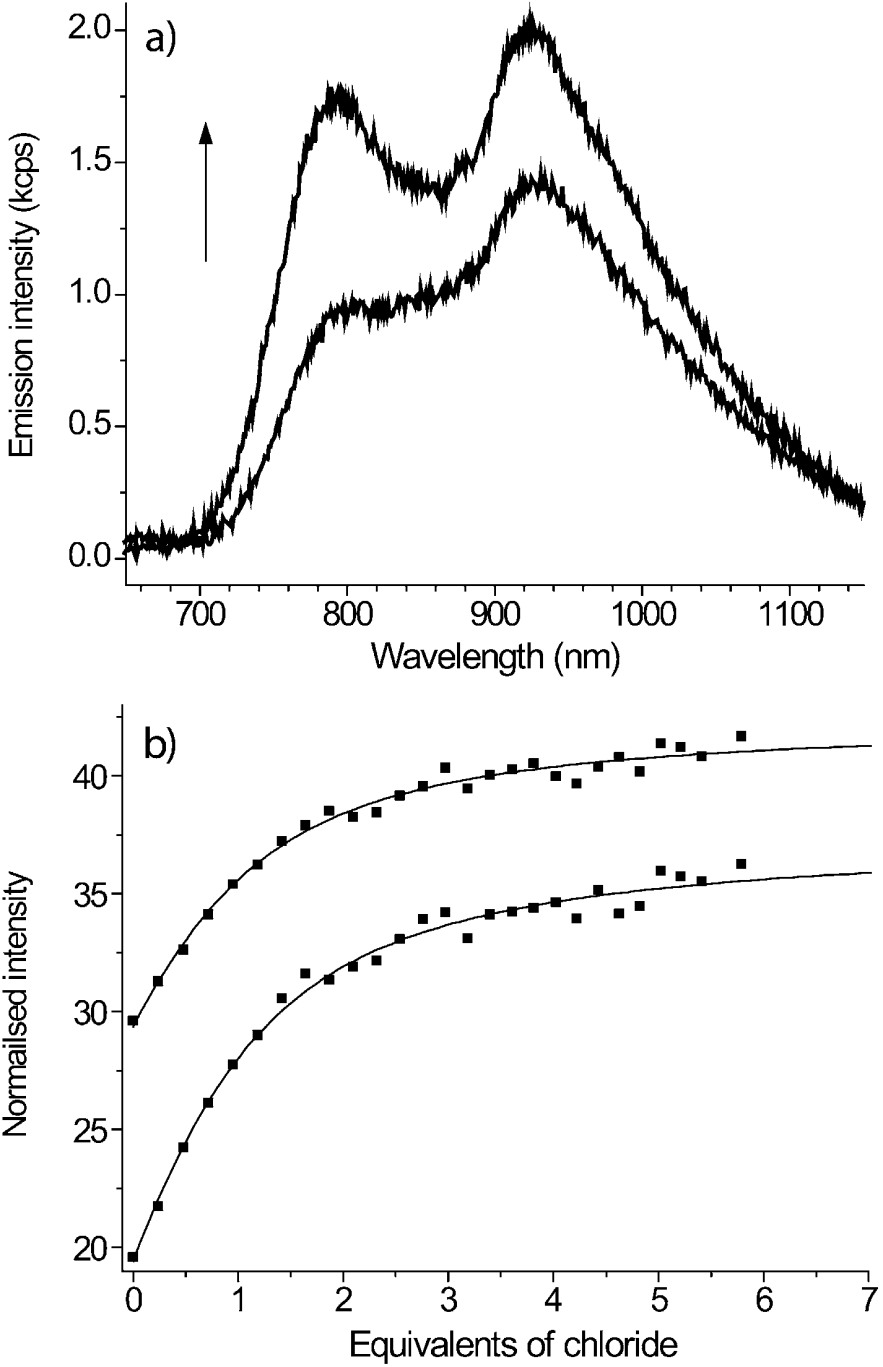
a) Titration of rotaxane 1 with TBACl in 97:3 acetonitrile/water, showing emission spectra uncorrected for detector sensitivity; (↑) increasing chloride anion concentration. b) Binding isotherms and fits from the same titration following the two emission bands.

**Luminescence studies**: The binding of anions was further investigated by using luminescence spectroscopy. Macrocycle **4** and the rotaxanes (**1**–**3**) all display two emission bands in the near-IR region centred at approximately 800 and 925 nm (Figure 4) following excitation into metal-to-ligand charge transfer (MLCT) absorption bands at 430 nm, with the lifetimes of the two bands determined to be 44 and 35 ns at 800 and 950 nm, respectively.

These observations are consistent with those of Nozaki and co-workers,[8] who assigned the presence of two bands in the emission spectra of osmium bipyridyl complexes to subtle variations in the osmium environment arising from solvation-induced polarisation of the triplet state. Upon titration with TBACl in 97:3 acetonitrile/water, an increase in emission intensity was observed for all four species, with the increases being more pronounced for the higher-energy bands. Given the conclusions of Nozaki regarding solvent-induced distortions to the triplet-state structure, it is plausible that anion binding will also influence local structure to differing degrees. Representative spectra are shown in Figure 4 along with the corresponding binding isotherms and fits. Association constants were obtained with the Dynafit software using a 1:1 binding model (Table 2).[11]

**Table 2:** Association constants [m^−1^] determined by luminescence titration of macrocycle 4 and rotaxanes 1, 2 and 3 in 97:3 acetonitrile/water, with chloride, dihydrogen phosphate and acetate anions, as TBA salts^[a]^

	Cl^−^	H_2_PO_4_^−^	AcO^−^
**1**	2.0×10^5^ [1.5–2.4×10^5^]	3.5×10^5^ [2.8–4.7×10^5^]	4.9×10^4^ [3.5–7.3×10^4^]
**2**	2.1×10^5^ [1.5–2.8×10^5^]	–^[b]^	–^[b]^
**3**	3.0×10^5^ [2.3–3.8×10^5^]	–^[b]^	–^[b]^
**4**	3.1×10^3^ [2.8–3.4×10^3^]	–^[c]^	2.4×10^3^ [2.2–2.6×10^3^]

[a] 99 % confidence intervals given in square brackets. A 1:1 binding model was used in all cases. [b] No data was obtained owing to insufficient material. [c] Could not be determined owing to precipitation.

Acetate and dihydrogen phosphate (as their TBA salts) were also titrated with rotaxane **1** and the macrocycle **4** for comparison, although with the latter, phosphate anion binding could not be determined owing to precipitation. The results are presented in Table 2. The data indicate that the binding of chloride anions with the rotaxanes is two orders of magnitude greater than that with the macrocycle. The chloride anion association constants obtained from luminescence measurements for the three rotaxanes confirm the observations from NMR spectroscopy of strong binding of chloride ions by rotaxane **3**. It should be noted that the change in solvent system will also be reflected in differences in binding owing to different solvation of the host. In this relatively uncompetitive solvent system, it is likely that many anions will associate with the cationic receptors; indeed, studies on dihydrogen phosphate and acetate bear out this hypothesis. It is important to point out that it is likely such optical titrations are notably less reflective of binding selectivity in that they report anion association, be it cavity confined or peripheral.

**Figure 5:**
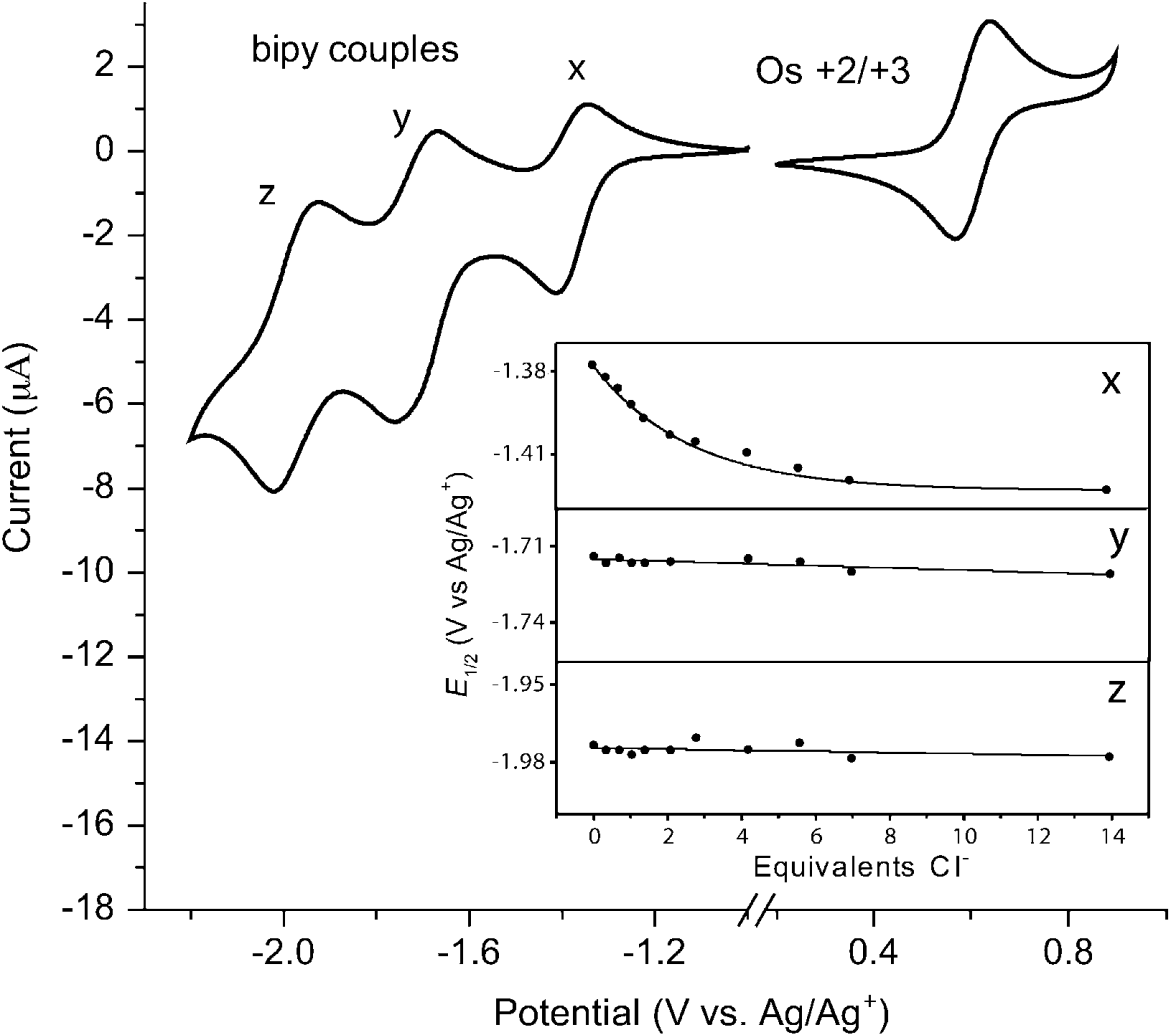
Cyclic voltammetric scans of 0.2 mm of macrocycle 4 in 0.15 m TBAPF_6_/CH_3_CN; scan rate=0.1 Vs^−1^. Insert: Electrochemical titrations of 4 with TBACl monitoring bipy couples x, y and z.

**Electrochemical investigations**: Electrochemical studies were initially carried out on macrocycle **4**. Cyclic voltammetric scans (Figure 5) of an acetonitrile solution containing macrocycle **4** revealed an electrochemical redox system at 0.605 V vs. Ag/Ag^+^, assigned to the reversible Os(+2/+3) redox couple.[12] In addition, the three bipyridyl-ligand-centred redox systems were observed at −1.375, −1.715 and −1.975 V vs. Ag/Ag^+^ (labelled x, y, z, respectively) consistent with previous reports of a tris(bipy) ruthenium moiety.[13] These assignments were confirmed by equivalent integration of the charges associated with the Os(+2/+3) redox system and the bipyridyl-ligand-centred redox events, as expected as all are one-electron redox systems on the same species. In accordance with our previous report,[13] the least-cathodic bipy redox couple (x, Figure 5) is assigned to the amide-substituted bipyridyl, located next to the anion-binding site (in light of the electron-withdrawing nature of the carbonyl amide moieties).

**Figure 6:**
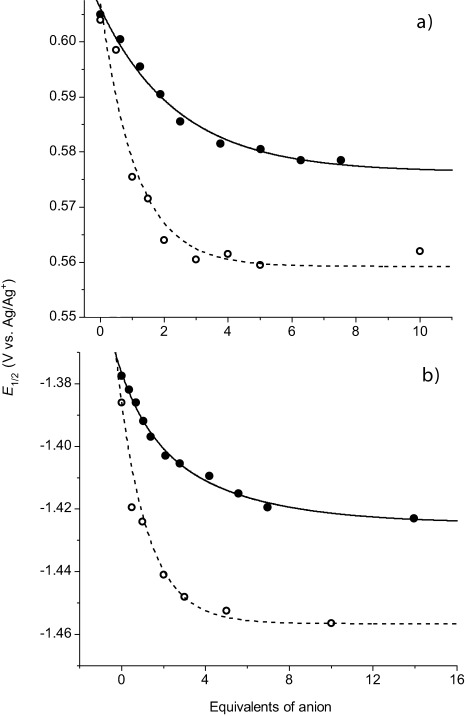
Redox transduction of cavity occupancy of compound 4. *E*_1/2_ of a) Os(+2/+3) and b) bipy couple versus equivalents of TBACl (•) and TBAAcO (○) added.

Titrations of macrocycle **4** with TBACl or TBAOAc were carried out to determine the ability of ligand- and metal-centre-based redox potentials to report on anion association. As shown in Figure 6 a, cathodic perturbations of approximately 20 mV and 40 mV of the metal-centred osmium-based (+2/+3) couple are observed upon the addition of ∼5 molar equivalents of TBACl and TBAOAc, respectively.[13] The bipy-ligand-centred responses scale in accordance with their vicinity to the anion-association site, with x showing the strongest perturbations and z the weakest, in the presence of 5-fold excess of Cl^−^ and AcO^−^ (see insert Figure 5, Schemes S2 and S3 in the Supporting Information). These observations are fully consistent with both anionic species being bound in the vicinity of the bis(amide) bipy cavity. Dynafit modelling analysis of a simultaneous fit of both the metal-centred Os(+2/+3) couple and the least-cathodic ligand-centred (x) potential perturbations as a function of anion concentration gave 1:1 stoichiometric association constants of 2.5×10^3^ [2.2–2.7×10^3^] and 1.5×10^4^ [1.1–2.1×10^4^] m^−1^, for chloride and acetate anions, respectively. The larger association constant (and potential shifts) observed after addition of TBAOAc, compared with TBACl, can be attributed to the stronger association of AcO^−^ to the amide-based binding site than Cl^−^, owing to the relative basicities of the anions.[13]

We assign the higher luminescence and electrochemically defined association constants, in comparison to those obtained by NMR spectroscopy, to the more competitive aqueous solvent system used for the latter, but we acknowledge also that optical and redox transitions may not be exactly equivalent in their response to specific anion association. Indeed, optical transition energies are likely to be sensitive to broad electrostatic/dielectric change in the vicinity of relevant chromophore orbitals; redox signatures are more specifically sensitive to electron density at the bipyridyl and osmium centres and, hence, may be more reflective of specific anion association. Resolved fundamental electrochemical characteristics of the rotaxane hosts are similar to those of the macrocycle and are reported in the Supporting Information (page 4). Unfortunately, a combination of sluggish diffusion and strong physical-adsorption-based electrode fouling precluded the attainment of reliable electrochemical anion-binding data with the rotaxanes.

**Scheme 3:**
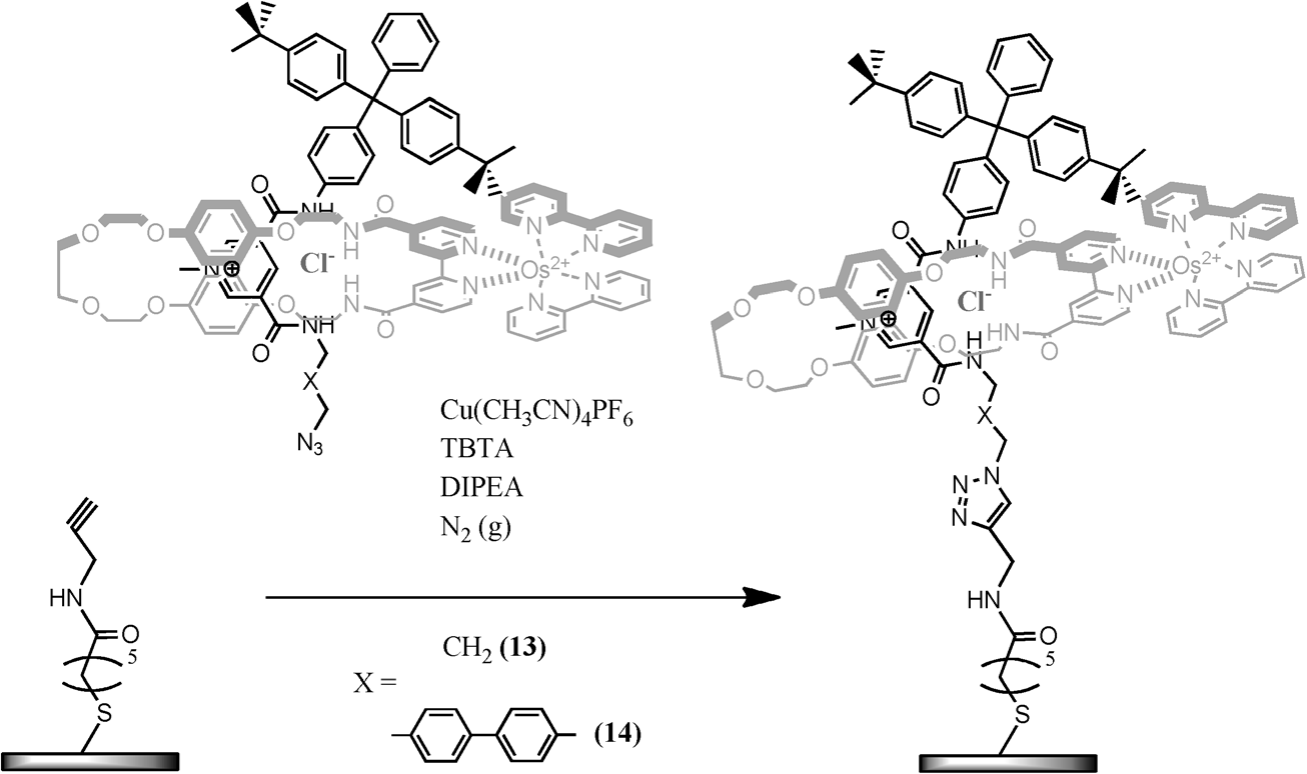
Rotaxane immobilisation onto alkyne-modified gold by copper(I)-catalysed Huisgen cycloaddition.

**Figure 7:**
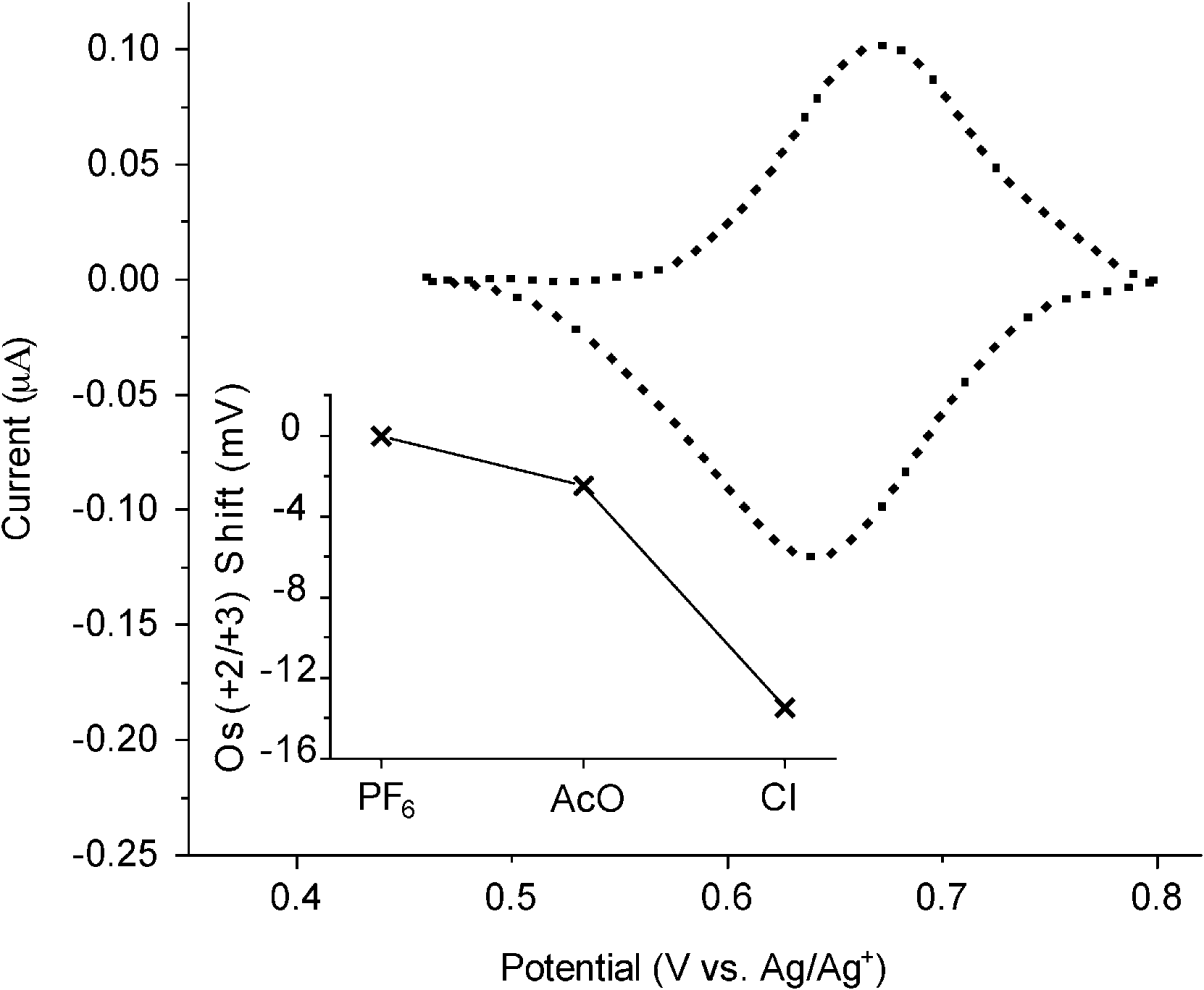
Cyclic voltammogram (background subtracted) signatures of rotaxane films based on 14. Stable over repeated cycling (>10 cycles). Estimated surface concentration as 50 % of theoretical maximum (calculated from a molecular footprint of the macrocycle, 3.0 nm^2^). Distance of faradaic exchange estimated at approximately 2 nm based on localisation of the macrocycle at the pyridinium station and a perpendicular orientation of the axle to the underlying gold surface. Insert: Potential shifts of surface-bound Os(+2/+3) couple, after immersion of rotaxane interface in TBAAcO/CH_3_CN and TBACl/CH_3_CN solutions following washing with NH_4_PF_6_/H_2_O to remove Cl^−^ template. Plotted data is the mean of several measurements.

**Surface immobilisation of the rotaxane**: The CuI-catalysed Huisgen cycloaddition of axles **13** and **14** to prepared alkyne-terminated molecular films on gold (see the Experimental Section) in the presence of templating chloride anions and a 5-fold excess of macrocycle **4**, generates surface-confined rotaxane assemblies (Scheme 3). Accompanying ellipsometry-defined increases in film thickness ((1.3±0.1) nm) are consistent with axle-to-surface orientations being normal or close to normal. Control surface analyses, in the absence of CuI catalyst, confirm this surface assembly to be specifically click-chemistry driven. The associated template locking of the osmium bipy macrocycle at the surface by this process is confirmed by resolved osmium-based (+2/+3) electrochemical signatures (Figure 7). An integration of these signals resolves surface macrocycle concentrations of 1.05 and 2.5×10^−11^ mol cm^−2^ for axles **13** and **14**, respectively, corresponding to 20 and 45 % of a densely packed monolayer, based on a macrocycle footprint of 3.0 nm^2^. Control experiments confirm that the macrocycle–surface association is both stable to ultrasonic washing and only observed with the chloride anion template specifically involved (importantly, no macrocycle-osmium-based (+2/+3) electrochemical response is observed if the PF_6_ salts of **13** or **14** are used). The linear scaling of Os(+2/+3) redox peak current with voltage scan rate is further confirmatory of surface entrapment. The surface-density differences between axle-only and rotaxane films are, additionally, resolvable through both electrode access of a solution-phase faradaic redox probe and surface-capacitance analysis; an axle-only film being significantly more densely packed (10 μF cm^−2^) than a rotaxane film with a “mushroom-shaped” component (25 and 15 μF cm^−2^ for surfaces prepared with **13** and **14**, respectively; Scheme S4 in the Supporting Information).

It is notable that axle **14** generates a significantly higher (>200 %) macrocycle capture on the surface than that observed with axle **13**, consistent with both increased axle rigidity and a higher associated chloride anion template association constant (Table 1). These films constitute not only an addition to the few surface-confined tethered interlocked systems but, to the best of our knowledge, are also the first incorporating a redox-active osmium(II) bipy reporter motif.

We have also investigated the possibility that surface-presented vacant interlocked cavities (generated by using axle **14**) can provide a means of selectively recruiting and electrochemically sensing chloride anions. The chloride anion template was initially removed by washing with copious amounts of 0.1 m NH_4_PF_6_/H_2_O. Pleasingly, subsequent halide anion recruitment is both detectable and selective, exhibiting no cathodic perturbation upon immersion in a 50 μm solution of acetate anions (the observed 2 mV shift is less than the 3 mV error of the reference electrode), but a 14 mV cathodic shift upon immersion in a chloride-anion-containing solution of the same concentration(with both solutions being formed from the TBA salt in anhydrous CH_3_CN, Figure 7). No potential shift was observed when the cavity was not depopulated prior to immersion in the chloride solution, as expected for a cavity exhibiting 1:1 binding stoichiometry.

These results are consistent with those presented in Table 1 and confirm the selectivity of the three-dimensional cavity towards chloride anion binding. Furthermore, the smaller potential shift observed here upon binding of chloride anions, in comparison with that observed of the macrocycle, is attributed to the electron-withdrawing nature of the positively charged pyridinium motif of the axle component, mitigating donation of electron density to the osmium centre, and is indicative of the interlocked surface-bound structure.

## Conclusion

Interlocked structures can be engineered to bind specific guests within the topologically constrained three-dimensional cavities created during their template-driven syntheses. This binding ability, when coupled to the signal-transduction capabilities associated with appended reporter groups, make catenanes and rotaxanes highly promising candidates for the development of molecular sensors.

We have reported herein the solution and surface synthesis of a number of anion-templated rotaxanes incorporating the photo- and electroactive tris(bipy) osmium moiety. Upon removal of the chloride anion template, ^1^H NMR titration of the solution rotaxanes with a number of anions (chosen on the basis of their contrasting size, shape and basicity) showed selectivity for chloride anions over acetate anions and dihydrogen phosphate oxoanions, observations broadly supported by associated perturbations in osmium luminescence or redox character. Rotaxane immobilisation onto an alkyne-modified gold electrode substrate by copper(I)-catalysed Huisgen cycloaddition produced molecular films capable of responding electrochemically and selectively to chloride anions. Notably, perturbation is only observed on prior cavity depopulation. This work clearly demonstrates the successful application of self-assembled monolayers to the electrochemical sensing of halide ions.

## Experimental Section

**General procedure**: Commercially available solvents and chemicals were used without further purification unless otherwise stated. Where dry solvents were used, they were degassed with nitrogen, dried by passing through an MBraun MPSP-800 column and then used immediately. Triethylamine was distilled from and stored over potassium hydroxide. Water was deionised and microfiltered by using a Milli-Q Millipore machine. ^1^H, ^13^C, ^19^F and ^31^P NMR spectra were recorded on a Varian Mercury-VX 300, and a Bruker AVII500 spectrometer. Mass spectrometry was carried out on a Bruker micrOTOF spectrometer.

**Synthesis**: Macrocycle **19**,[6b] axle **5**,[3a] bis(amine) **8**,[9] thread **17**,[3a] and axle **14**[14] have been synthesized according to reported procedures. The syntheses of **11**, **12** and **13** along with electrochemical, surface analysis and luminescence anion titration details are given in the Supporting Information.

**Rotaxane 1**: In a 50 mL round-bottom flask, 2,2′-bipyridine-4,4′-dicarboxylic acid (60 mg, 245 μmol, 1.2 equiv) was suspended in 10 mL of thionyl chloride, a drop of DMF was added and the solution was heated at reflux under N_2_ for 16 h. After removal of the solvent, the residue was dissolved in 10 mL of dry dichloromethane and added dropwise to a 50 mL dry dichloromethane solution containing bis(amine) **8** (86 mg, 204 μmol, 1 equiv), axle **5** (220 mg, 204 μmol, 1 equiv) and triethylamine (71 μL, 510 μmol, 2.5 equiv). The reaction mixture was stirred at room temperature (21±3 °C) for 2 h, washed with 10 % HCl_(aq.)_ (2×50 mL) and water (2×50 mL) and dried over MgSO_4_. After removal of the solvent, the residue was dissolved in 10 mL of acetonitrile, filtered and solvent removed in vacuo to give 121 mg of a crude mixture containing rotaxane and macrocycle **19**. This crude material (121 mg, 71 μmol, 1 equiv) was suspended in 20 mL of a 4:1 EtOH/H_2_O mixture and [Os(bipy)_2_Cl_2_] **16** (82 mg, 142 μmol 2 equiv) was added. The solution was heated at reflux for 16 h under N_2_. After removal of the solvent, the crude product was purified by preparative TLC (SiO_2_, CH_3_CN/H_2_O/KNO_3(sat. aq.)_ 14:2:1). Rotaxane **1** (30 mg, 6 %) was obtained as a brown solid after anion exchange to the hexafluorophosphate salt by washing a dichloromethane solution of the rotaxane with 0.1 m NH_4_PF_6(aq.)_ (8×15 mL) and H_2_O (2×15 mL). ^1^H NMR (500 MHz, [D_6_]acetone/D_2_O (7:3)): *δ*=9.40 (2 H, s; Py_β_), 9.33 (1 H, s; Py_γ_), 9.29 (2 H, s; ArH_c_), 8.75 (2 H, d, ^3^*J*=8.4 Hz; bipy), 8.71 (2 H, d, ^3^*J*=8.4 Hz; bipy), 8.05 (2 H, d, ^3^*J*=6.2 Hz; ArH_a_), 8.02 (2 H, t, ^3^*J*=8.1 Hz; bipy), 7.90 (2 H, t, ^3^*J*=8.1 Hz; bipy), 7.78 (2 H, d, ^3^*J*=5.6 Hz; bipy), 7.71 (2 H, d, ^3^*J*=5.6 Hz; bipy), 7.66 (2 H, dd, ^3^*J*=6.1.4 Hz, ^4^*J*=1.7 Hz; ArH_b_), 7.59 (4 H, s, ^3^*J*=8.9 Hz; ArH_ε_), 7.46 (2 H, t, ^3^*J*=6.7 Hz; bipy), 7.06–7.27 (28 H, m; ArH_stopper_ and bipy), 6.50 (4 H, m, ^3^*J*=8.9 Hz; ArH_g_), 6.39 (4 H, m, ^3^*J*=9.1 Hz; ArH_h_), 4.61 (3 H, s; H_α_), 3.92 (4 H, m; CH_2_), 3.86 (4 H, m; CH_2_), 3.79 (4 H, m; CH_2_), 3.77 (4 H, s; CH_2_), 3.54 (4 H, m; CH_2_), 1.18 ppm (36 H, s; *t*BuH); ^13^C NMR (125 MHz, [D_6_]acetone): *δ*=164.0, 160.7, 159.7, 159.5, 152.4, 151.7, 149.5, 147.9, 144.7, 138.9, 138.8, 132.3, 131.7, 131.4, 129.4, 129.3, 128.4, 127.4, 126.9, 125.7, 125.7, 125.3, 123.2, 121.1, 116.5, 115.4, 71.4, 70.9, 67.5, 64.7, 40.4, 34.9, 31.7 ppm; ^19^F NMR (282.5 MHz, [D_6_]acetone): *δ*=−72.4 ppm (d, ^1^*J*=707 Hz; PF_6_); ^31^P NMR (121.6 MHz, [D_6_]acetone): *δ*=−144.3 ppm (sept, ^1^*J*=707 Hz; PF_6_); MS (MALDI-TOF): *m*/*z* calcd for [C_128_H_130_F_18_N_11_O_10_OsP_3_] [*M*−H−3(PF_6_)]^2+^: 1085.98; found: 1085.98.

**Rotaxane 2**: In a 50 mL round-bottom flask, osmium macrocycle **4** (100 mg, 70 μmol, 2 equiv) and thread **13** (26 mg, 35 μmol, 1 equiv) were slowly stirred for 1 h in 25 mL of dry dichloromethane. After removal of the solvent, the residue was dissolved in 25 mL of dry and degassed dichloromethane. Stopper **15** (19 mg, 35 μmol, 1 equiv), Cu(CH_3_CN)_4_PF_6_ (2.6 mg, 7 μmol, 0.2 equiv), TBTA (3.7 mg, 7 μmol, 0.2 equiv) and diisopropylethylamine (DIPEA) (12 μL, 70 μmol, 2 equiv) were successively added and the reaction mixture stirred at room temperature (21±3 °C) for 3 d. Then, 10 mL of KNO_3(sat. aq.)_ was added, vigorously stirred for 30 min and the organic layer was extracted. After removal of the solvent, the crude product was purified by preparative TLC (SiO_2_, CH_3_CN/H_2_O/KNO_3(sat. aq.)_ 14:2:1). Rotaxane **2** (16 mg, 16 %) was obtained as a brown solid after anion exchange to the hexafluorophosphate salt by washing a chloroform solution of the rotaxane with 0.1 m NH_4_PF_6(aq.)_ (8×15 mL) and H_2_O (2×15 mL). ^1^H NMR (500 MHz, [D_6_]acetone): *δ*=10.06 (1 H, s; NH_d_), 9.42 (1 H, s; Py_g_), 9.36 (3 H, m; Py_b/b′_, ArH_c_ and ArH_c′_), 9.20 (1 H, s; Py_b/b′_), 8.82 (2 H, d, ^3^*J*=7.5 Hz; bipy), 8.77 (2 H, d, ^3^*J*=8.2 Hz; bipy), 8.74 (2 H, d, ^3^*J*=8.2 Hz; bipy), 8.36 (1 H, s; NH_d′_), 8.20 (2 H, m; ArH_a_), 8.04 (1 H, s; H_c_), 7.96–8.03 (8 H, m; bipy), 7.78, (2 H, d, ^3^*J*=6.1 Hz; ArH_b_*J*), 7.74 (2 H, d, ^3^*J*=5.8 Hz; ArH_e_), 7.51 (4 H, m; bipy), 7.14–7.39 (29 H, m; ArH_stopper_), 6.90 (2 H, d, ^3^*J*=8.8 Hz; ArH_e′_), 6.63 (4 H, s; ArH_g_), 6.47 (4 H, s; ArH_h_), 5.03 (2 H, s; H_l_), 4.65 (3 H, s; H_a_), 4.44 (2 H, t, ^3^*J*=6.8 Hz; H_n_*J*), 4.04 (4 H, m; OCH_2_), 3.99 (4 H, m; OCH_2_), 3.91 (8 H, s; NCH_2_ and OCH_2_), 3.63 (4 H, m; OCH_2_), 3.48 (2 H, m; H_r_), 2.19 (2 H, m; H_k_), 1.29 (18 H, s; *t*BuH), 1.27 ppm (27 H, s; *t*BuH); ^13^C NMR (125 MHz, [D_6_]acetone): *δ*=164.4, 160.8, 159.8, 159.7, 159.6, 157.4, 153.3, 152.5, 152.4, 152.0, 151.7, 149.6, 149.3, 148.0, 145.3, 144.8, 144.2, 142.3, 140.8, 138.9, 132.9, 132.4, 131.8, 131.5, 131.4, 129.6, 129.4, 128.5, 127.2, 126.9, 125.7, 125.4, 125.2, 125.2 *sic*, 125.1, 123.4, 121.0, 120.9, 116.6, 115.5, 115.5 *sic*, 114.3, 71.5, 70.9, 68.5, 67.5, 67.4, 64.8, 64.0, 62.2, 55.0, 50.2, 48.5, 40.3, 36.2, 34.9, 31.7 ppm; ^19^F NMR (282.5 MHz, [D_6_]acetone): *δ*=−72.5 ppm (d, ^1^*J*=708 Hz; PF_6_); ^31^P NMR (121.6 MHz, [D_6_]acetone): *δ*=−144.3 ppm (sept, ^1^*J*=709 Hz; PF_6_); MS (MALDI-TOF): *m*/*z* calcd for [C_138_H_147_F_18_N_14_O_11_OsP_3_] [*M*−(PF_6_)]^+^: 2658.03; found: 2658.26.

**Rotaxane 3**: In a 50 mL round-bottom flask, osmium macrocycle **4** (100 mg, 70 μmol, 2 equiv) and thread **14** (31 mg, 35 μmol, 1 equiv) were slowly stirred for 1 h in 25 mL of dry dichloromethane. After removal of the solvent, the residue was dissolved in 25 mL of dry and degassed dichloromethane. Stopper **15** (19 mg, 35 μmol, 1 equiv), Cu(CH_3_CN)_4_PF_6_ (2.6 mg, 7 μmol, 0.2 equiv), TBTA (3.7 mg, 7 μmol, 0.2 equiv) and DIPEA (12 μL, 70 μmol, 2 equiv) were successively added and the reaction mixture was stirred at room temperature (21±3 °C) for 3 d. Then, 10 mL of KNO_3(sat. aq.)_ was added, vigorously stirred for 30 min and the organic layer was extracted. After removal of the solvent, the crude product was purified by preparative TLC (SiO_2_, CH_3_CN/H_2_O/KNO_3(sat. aq.)_ 14:2:1). Rotaxane **3** (74 mg, 72 %) was obtained as a brown solid after anion exchange to the hexafluorophosphate salt by washing a chloroform solution of the rotaxane with 0.1 m NH_4_PF_6(aq.)_ (8×15 mL) and H_2_O (2×15 mL). ^1^H NMR (500 MHz, [D_6_]acetone): *δ*=9.92 (1 H, s; NH_δ_), 9.44 (1 H, s; Py_γ_), 9.34 (1 H, s; Py_β/β′_), 9.27 (1 H, s; ArH_c/c′_), 9.23 (1 H, s; Py_β/β′_), 9.12 (1 H, s; ArH_c/c′_), 8.78 (4 H, d, ^3^*J*=8.5 Hz; bipy), 8.36 (1 H, s; NH_δ′_), 8.18 (2 H, d, ^3^*J*=6.1 Hz; ArH_a_), 8.17 (1 H, s; H_χ_), 8.03 (4 H, m; bipy), 7.87 (4 H, m; bipy), 7.80 (2 H, m; ArH_b_), 7.70 (2 H, s; ArH_ε_), 7.48 (4 H, m; bipy), 7.09–7.42 (37 H, m; ArH_stopper_ and ArH_biphenyl_), 6.92 (2 H, d, ^3^*J*=9.0 Hz; ArH_ε′_), 6.61 (4 H, m; ArH_g_), 6.41 (4 H, m; ArH_h_), 5.67 (2 H, s; H_ν_), 5.16 (2 H, s; H_λ_), 4.67 (3 H, s; H_α_), 4.08 (4 H, m; OCH_2_), 4.04 (4 H, m; OCH_2_), 3.94 (4 H, m; OCH_2_), 3.87 (8 H, m; NCH_2_ and OCH_2_), 3.71 (2 H, s; H_ρ_), 1.30 (18 H, s; *t*BuH), 1.28 ppm (27 H, s; *t*BuH); ^13^C NMR (125 MHz, [D_6_]acetone): *δ*=163.8, 162.4, 160.3, 159.4, 159.1, 157.1, 152.8, 152.2, 151.9, 151.4, 151.3, 151.2, 149.1, 148.8, 147.6, 146.4, 144.9, 144.3, 141.8, 140.3, 138.5, 137.6, 135.8, 132.4, 132.0, 131.3, 131.1, 131.0, 129.2, 129.1, 129.0, 128.7, 128.1, 127.6, 127.5, 127.0, 126.5, 125.3, 125.0, 124.7, 123.2, 122.7, 120.5, 116.3, 116.2, 115.0, 113.9, 71.0, 70.4, 68.0, 67.2, 67.0, 64.4, 63.4, 61.9, 54.6, 53.6, 49.7, 39.8, 35.8, 34.6, 34.5, 31.3 ppm;^19^F NMR (282.5 MHz, [D_6_]acetone): *δ*=−72.5 ppm (d, ^1^*J*=708 Hz; PF_6_); ^31^P NMR (121.6 MHz, [D_6_]acetone): *δ*=−144.3 ppm (sept, ^1^*J*=709 Hz; PF_6_); MS (MALDI-TOF): *m*/*z* calcd for [C_149_H_153_F_18_N_14_O_11_OsP_3_] [*M*−(PF_6_)]^+^: 2796.07; found: 2796.10.

**Macrocycle 4**: In a 250 mL round-bottom flask, macrocycle **19** (168 mg, 270 μmol, 1 equiv) was suspended in 100 mL of a 4:1 EtOH/H_2_O mixture and [Os(bipy)_2_Cl_2_] **16** (153 mg, 142 mg, 2 equiv) was added. The solution was heated at reflux for 36 h under N_2_. The brown solution was left to cool to room temperature (21±3 °C) and then filtered through a Celite bed before the solvent was removed in vacuo. The brown residue was then redissolved in 20 mL of H_2_O and NH_4_PF_6(s)_ was added to the solution until precipitation ceased. The precipitate was collected by vacuum filtration and dried under vacuum to give macrocycle **4** as a black solid (282 mg, 198 μmol, 74 %). ^1^H NMR (300 MHz, [D_6_]acetone): *δ*=9.14 (2 H, s; ArH_c_), 8.82 (4 H, m; bipy), 8.51 (2 H, t, ^3^*J*=5.3 Hz; NH_d_), 8.19 (2 H, d, ^3^*J*=5.6 Hz; ArH_a_), 8.05 (4 H, t, ^3^*J*=7.9 Hz; bipy), 7.97 (4 H, t, ^3^*J*=6.0 Hz; bipy), 7.80 (2 H, dd, ^3^*J*=6.1 Hz, ^4^*J*=1.7 Hz; ArH_b_), 7.53 (2 H, t, ^3^*J*=6.8 Hz; bipy), 7.47 (2 H, t, ^3^*J*=6.8 Hz; bipy), 6.88 (8 H, m; ArH_g,h_), 4.14 (4 H, t, ^3^*J*=5.3 Hz; CH_2_), 4.06 (4 H, t, ^3^*J*=4.5 Hz; CH_2_), 3.73–3.85 (8 H, m; CH_2_), 3.67 ppm (4 H, s; CH_2_); ^13^C NMR (75.5 MHz, [D_6_]acetone): *δ*=163.6, 160.8, 159.7, 153.9, 152.4, 152.2, 151.7, 142.4, 138.8, 129.4, 127.5, 125.6, 122.7, 116.5, 116.3, 71.4, 70.4, 68.9, 67.4, 40.5 ppm; ^19^F NMR (282.5 MHz, [D_6_]acetone): *δ*=−72.5 ppm (d, ^1^*J*=708 Hz; PF_6_); ^31^P NMR (121.6 MHz, [D_6_]acetone): *δ*=−144.3 (sept, ^1^*J*=709 Hz; PF_6_); MS (MALDI): *m*/*z* calcd for [C_54_H_52_F_12_N_8_O_8_OsP_2_] [*M*−(PF_6_)_2_]^2+^: 1132.35; found: 1132.38.

## Supporting Information

As a service to our authors and readers, this journal provides supporting information supplied by the authors. Such materials are peer reviewed and may be re-organized for online delivery, but are not copy-edited or typeset. Technical support issues arising from supporting information (other than missing files) should be addressed to the authors.

miscellaneous_information
