# Supplementary material for: Anion Sensing by Solution- and Surface-Assembled Osmium(II) Bipyridyl Rotaxanes
Source: Chemistry. 2013 Oct 14;19(47):15898–906. doi: 10.1002/chem.201302886 (PMC4517173; doi:10.1002/chem.201302886)
Supplement: Supplementary file 1 — miscellaneous_information [file chem0019-15898-sd1.pdf]

CHEMISTRY
A EUROPEAN JOURNAL

Supporting Information

© Copyright Wiley-VCH Verlag GmbH & Co. KGaA, 69451 Weinheim, 2013

Anion Sensing by Solution- and Surface-Assembled Osmium(II) Bipyridyl Rotaxanes

Joshua Lehr, Thomas Lang, Octavia A. Blackburn, Timothy A. Barendt, Stephen Faulkner, Jason J. Davis,* and Paul D. Beer*[a]

chem_201302886_sm_miscellaneous_information.pdf

Experimental Details

Synthesis

Compound 11: In a 50 mL round-bottom flask, compound **10** (400 mg, 670 µmol, 1 eq.) was suspended in 25 mL of dry dichloromethane, oxalyl chloride (120 µL, 1.34 mmol, 2 eq.) and DMF (catalytic amount) were added and stirred at room temperature under N_2 for 2h. Solvent was removed in vacuum and residue redissolved in 25 mL of dry dichloromethane. The latter was added dropwise to 3-bromopropylamine hydrobromide (147 mg, 670 µmol, 1 eq.) and triethylamine (280 µL, 2.01 mmol, 3 eq.) in solution in 50 mL of dry dichloromethane and stirred at room temperature under N_2 for 2h. The reaction mixture was then washed with 10% HCl $_{(aq.)}$ (2x100 mL) and water (2x 100 mL), dried over $MgSO_4$ and solvent removed in vacuum. Crude product was purified by chromatography (SiO_2, CH_2Cl_2/MeOH, 1/0 to 95/5) yielding to compound **11** (160 mg, 33%) as a pale orange solid.
^{1}H NMR (300 MHz, $CDCl_3$) δ (ppm): 9.19 (1H, s, Py), 9.12 (1H, s, Py), 9.07 (1H, s, Py), 8.72 (1H, s, NH), 7.58 (2H, d, ArH, ^3J = 8.8 Hz), 7.40 (1H, d, NH, ^3J = 5.5 Hz), 7.12 – 7.30 (15H, m, $ArH_{stopper}$), 3.59 (2H, q, NCH_2, ^3J = 6.4 Hz), 3.44 (2H, t, $BrCH_2$, ^3J = 6.3 Hz), 2.17 (2H, m, CH_2, ^3J = 6.5 Hz), 1.33 (18H, s, *t*-BuH). ^{13}C NMR (75 MHz, $CDCl_3$) δ (ppm): 148.6, 147.1, 147.0, 144.9, 144.2, 143.7, 143.6, 135.4, 135.0, 131.9, 131.8, 131.1, 130.7, 127.5, 125.9, 124.5, 119.7, 63.9, 34.4, 31.5, 28.0. MS (ESI): *m/z* calc. for $C_{43}H_{46}BrN_3O_2$ $[M + H]^+$: 718.28; found: 718.29.

Compound 12: In a 50 mL round-bottom flask, compound **11** (160 mg, 223 µmol, 1 eq.) was dissolved in 20 mL of dry and degassed DMF. Sodium azide (22 mg, 335 µmol, 1.5 eq.) was added and the reaction mixture stirred at 90°C under N_2 for 16h. After removal of the solvent, the crude was dissolved in 20 mL of dichloromethane, washed with water (3x20 mL). The aqueous layers were combined and washed with dichloromethane (3x20 mL) and the combined organic layer was dried over $MgSO_4$. Solvent was removed leading to the desired compound **12** (148 mg, 98%) as a white solid.
^{1}H NMR (300 MHz, $CDCl_3$) δ (ppm): 9.24 (1H, s, Py), 9.19 (1H, s, Py), 9.10 (1H, s, Py), 8.62 (1H, s, NH), 7.59 (2H, d, ArH, ^3J = 8.8 Hz), 7.53 (1H, d, NH, ^3J = 5.8 Hz), 7.12 – 7.30 (15H, m, $ArH_{stopper}$), 3.53 (2H, q, NCH_2, ^3J = 6.3 Hz), 3.41 (2H, t, N_3CH_2, ^3J = 6.5 Hz), 1.89 (2H, m, CH_2, ^3J = 6.7 Hz), 1.33 (18H, s, *t*-BuH). ^{13}C NMR (75 MHz, $CDCl_3$) δ (ppm): 165.5, 163.8, 150.8, 150.3, 148.6, 147.1, 144.4, 143.6, 135.4, 134.3, 131.9, 131.2, 131.0, 130.7, 130.0, 127.5, 125.9, 124.4, 119.6, 63.9, 49.2, 38.0, 34.4, 31.5, 28.6. MS (ESI): *m/z* calc. for $C_{43}H_{46}N_6O_2$ $[M + Na]^+$: 701.36; found: 701.37.

Thread 13: In a 25 mL round-bottom flask, compound **12** (148 mg, 218 µmol) was dissolved in 10 mL of methyl iodide and heated at reflux under N_2 for 16h. After removal of the solvent, the crude yellow solid was redissolved in 15 mL of dichloromethane and washed with 1 M NH_4Cl $_{(aq.)}$ (8x15 mL) and water (2x15 mL), dried over $MgSO_4$. After removal of the solvent, the desired chloride thread **13** (120 mg, 76%) was obtained as a yellow solid.
^{1}H NMR (300 MHz, $CDCl_3$) δ (ppm): 10.64 (1H, s, Py), 10.35 (1H, s, NH), 9.38 (2H, m, Py and NH), 8.32 (1H, s, Py), 7.71 (2H, d, ArH, ^3J = 8.6 Hz), 7.16 – 7.34 (15H, m, $ArH_{stopper}$), 3.86 (3H, s, CH_3), 3.48 (4H, m, N_3CH_2 and NCH_2), 1.95 (2H, m, CH_2, ^3J = 6.5 Hz), 1.29 (18H, s, *t*-BuH). ^{13}C NMR (75 MHz, $CDCl_3$) δ (ppm): 160.4, 158.1, 148.6, 147.6, 147.3, 146.1, 145.3, 143.9, 141.2, 135.2, 134.2, 133.7, 131.6, 131.0, 130.5, 127.8, 125.9, 124.7, 119.4, 64.1, 49.1, 37.9, 34.4, 31.5, 28.3. MS (ESI): *m/z* calc. for $C_{44}H_{49}ClN_6O_2$ $[M - Cl]^+$: 693.39; found: 693.34.

Ellipsometry

Ellipsometry was carried out using Beaglehole Instruments Picometer Ellipsometer assuming a refractive index of 1.45 for the monolayer over the gold layer (with pseudo optical constants of approximately n+ik =0.52+i×3.570 determined from a fit to each individual gold sample prior to modification). The given ellipsommetry film thicknesses are averages of four measurements over two separately prepared samples, with two measurements per sample; the error was determined from the range of the four values.

Electrochemistry

Electrochemistry was performed on a Autolab PGSTAT-12 system and data was analysed using General Purpose Electrochemical Software (GPES) version 4.9. All electrochemistry was undertaken was undertaken in anhydrous acetonitrile with 0.15 M $TBAPF_6$ supporting electrolyte. Acetonitrile was chosen due to its large potential window (allowing for the observation of bipy electrochemistry) and high solubility of the macrocycle in this medium. Silver/silver nitrate reference electrode and platinum wire auxiliary were used. Diffusive voltammetry of macrocycle **4** was undertaken at a 3mm diameter boron doped diamond (Windsor scientific) working electrode; a concentration of 0.2 mM macrocycle was used.

Electrochemical titrations of **4** were carried out by additions of 12.5 mM solution of the TBA salt of the analyte in 0.15 M $TBAPF_6$/acetonitrile. The potential of the reference electrode was compared to ferrocene before and after each titration to ensure a stable reference potential in place for the course of the titration. Anion recruitment at rotaxane surfaces was investigated by observation of the Os (+2/+3) oxidation signal with square wave voltammetry after immersion of the electrode in a 50 µM solution of the relevant salt for 30 minutes, followed by washing (10 seconds) in anhydrous acetonitrile.

NMR Titrations

The NMR titrations were carried out with the following initial concentrations of receptors: 1 Cl: $1.89.10^{-3}$; 1 OAc: $1.89.10^{-3}$; 2 Cl: $2.85.10^{-3}$; 3 Cl: $1.81.10^{-3}$; 4 Cl: $2.76.10^{-3}$ $mol.L^{-1}$.

Preparation of gold substrates

All polycrystalline gold disk working electrodes (diameter = 1.6 mm) on which the surface bound rotaxanes were studied were purchased from BASi. After initial mechanical polishing (0.3 µm alumina) and subsequent sonication, electrodes were immersed in fresh piranha solution (a 3:1 by volume mixture of 18 M sulphuric acid and 30% hydrogen peroxide) for 5 minutes, prior to electrochemical polishing by recording cyclic voltammograms in 0.5 M sulphuric acid in the potential region of the gold/gold oxide system. The surface area of the electrodes was determined from the gold/gold oxide redox system was described previously.[1]

Gold substrates for ellipsometry were prepared by vapour deposition of 10 nm of chromium, followed by 100 nm of gold, onto Si (100) substrates (Silicon Materials). Prior to modification the substrates were cleaned by immersion in piranha solution for 10 seconds followed by rinsing with copious amounts of millipore water and drying by a stream of N_2 (g).

Preparation of alkyne films

Gold electrodes and substrates were modified with carboxylic acid terminated SAMs by immersion 6-mercapohexanoic acid (MHA, sigma-aldrich) in ethanol (HPLC grade, sigma-aldrich) for > 12 hours. After rinsing with ethanol and drying with N_2 the samples were transferred to 0.4 M ethyl(dimethylaminopropyl) carbodiimide, 0.1 M N-Hydroxysuccinimide / H_2O for 40 mins prior to rinsing and immersion in 0.1 M propargylamine / H_2O for 24 hours.

Surface "Click" protocol

The attachment of axles **13** and **14** (in the presence **4**) was undertaken by cycloaddition. Gold substrate and electrodes were immersed in a solution of 0.1 mM azide axle, 0.2 mM TBTA, 0.2 mM $Cu(CH_3CN)_4PF_6$, 40 mM DIPEA and 0.5 mM macrocycle **4**, in oxygen free atmosphere.

Synthesis

The synthesis of the three rotaxanes from their immediate components is described in **SI 1** below.

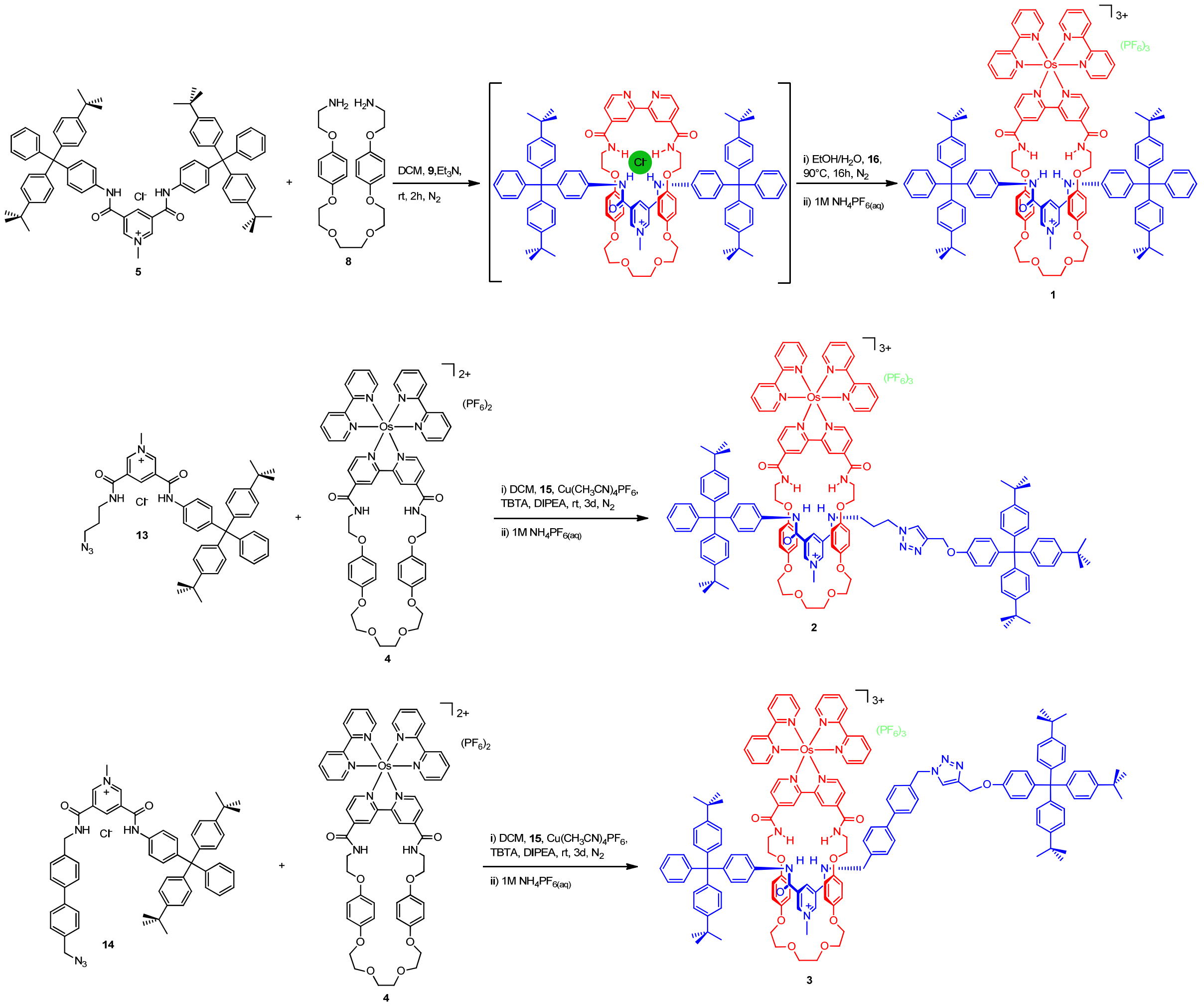

SI 1. Synthesis of rotaxanes **1**, **2**, and **3** by clipping (**1**) and stoppering (**2** and **3**) anion templation strategies. After preparation of the rotaxane the Cl^- template was removed by washing with NH_4PF_6/H_2O giving the PF_6 salt product.

Electrochemistry

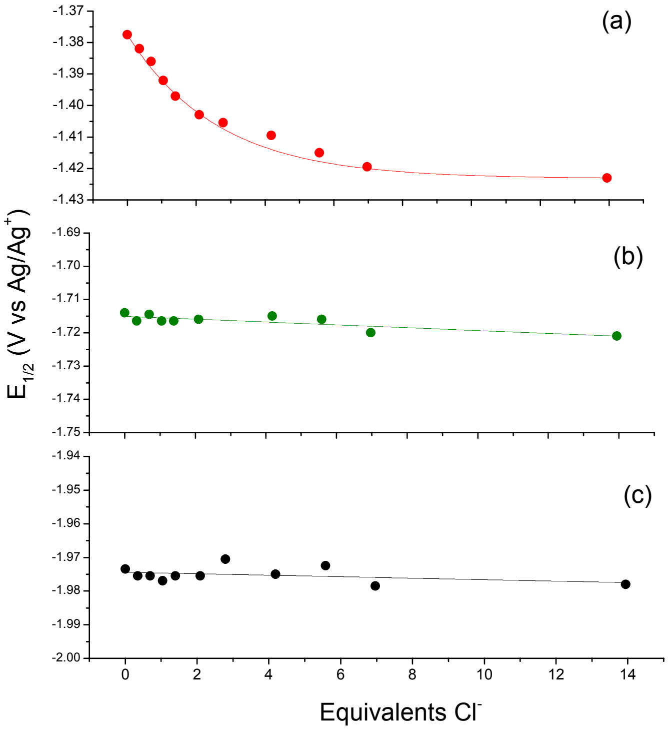

SI 2. Plot of $E_{1/2}$ of bipy *x* (a) *y* (b) and *z* (c) redox couples vs. additions of equivalents of TBACl for macrocycle **4**.

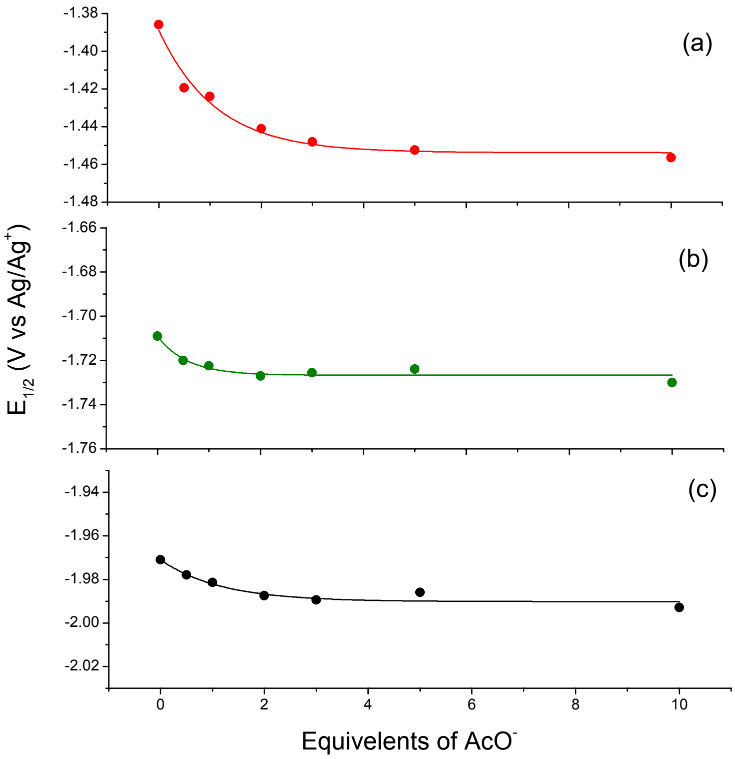

SI 3 . Plot of $E_{1/2}$ of bipy *x* (a) *y* (b) and *z* (c) redox couples vs. additions of equivalents of TBAAcO for macrocycle **4**.

Electrochemistry rotaxanes **1** and **3** revealed the Os (+2/+3) redox couple at $E_{1/2}$ = 0.595 and 0.565 V vs. SCE respectively. Reliable voltametric signals for the bipy centred couples could not be obtained.

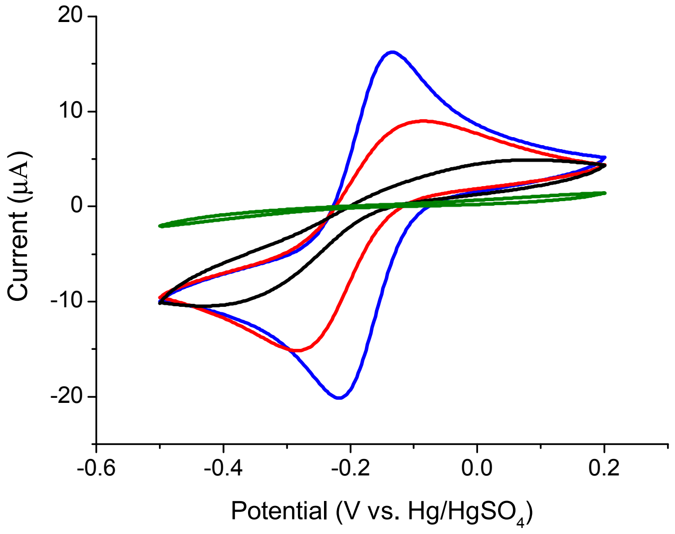

SI 4. Cyclic voltammograms in the presence of $Fe(CN)_6^{3-/4-}$ of bare gold (blue), alkyne modified gold (red) the alkyne modified gold after click reaction of the pseudorotaxne formed from **13** and **4** (black) and alkyne modified gold after click reaction of the PF_6 salt of **13** in the presence of **4** (green).

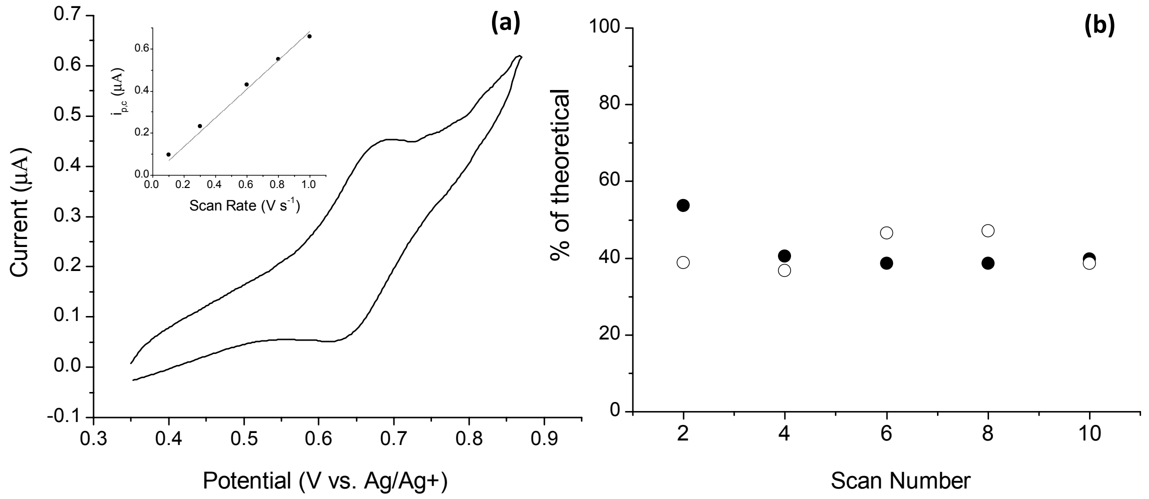

SI 5. (a) Cyclic voltammogram of alkyne modified electrode after Huisgien cycloaddition of axle **14** in the presence of macrocycle **4** (insert) scan rate vs. cathodic peak current. (b) % of theoretical surface concentration from charge of oxidation (○) and reduction (●).

Luminescence Studies

Changes in luminescence intensity upon titration of **1**, **2**, **3** and **4** with anions are shown below with binding isotherms and fits.

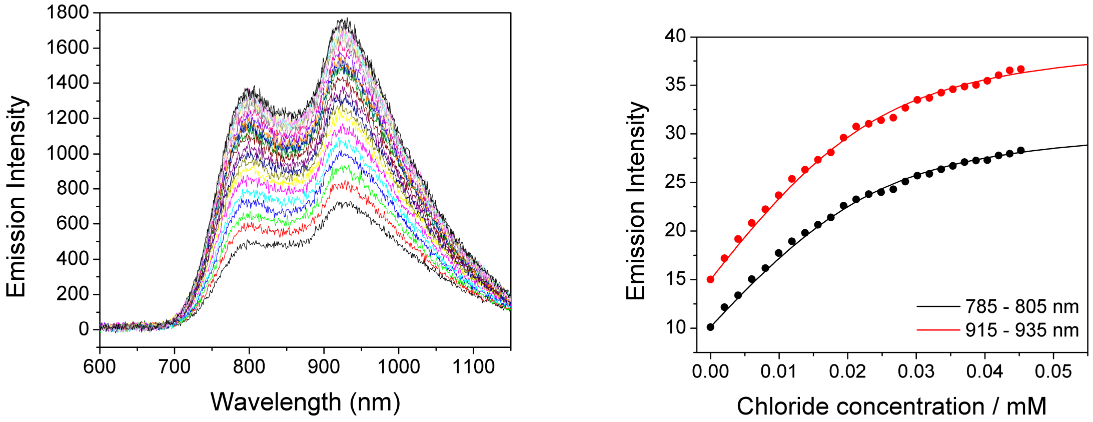

SI 6. Titration of a 2×10^{-5} M solution of rotaxane **2** with TBA.Cl in 97:3 acetonitrile: water. *Left:* showing the changes in the emission spectra upon 430 nm excitation. *Right:* the binding isotherms for Os(bipy)$_3$-based emission bands upon 430 nm excitation: in each case, the line shows the best fit obtained.

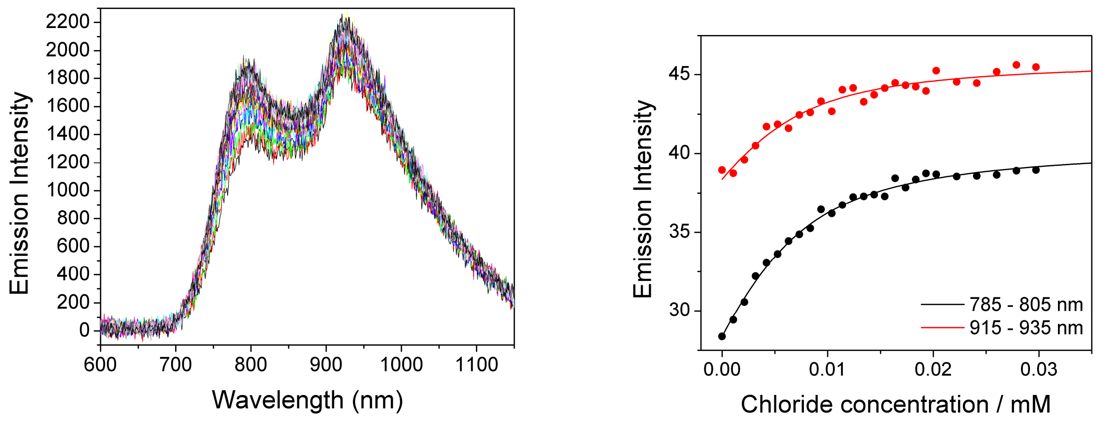

SI 7. Titration of a 2×10^{-5} M solution of rotaxane **3** with TBA.Cl in 97:3 acetonitrile: water. *Left:* showing the changes in the emission spectra upon 430 nm excitation. *Right:* the binding isotherms for Os(bipy)$_3$-based emission bands upon 430 nm excitation: in each case, the line shows the best fit obtained.

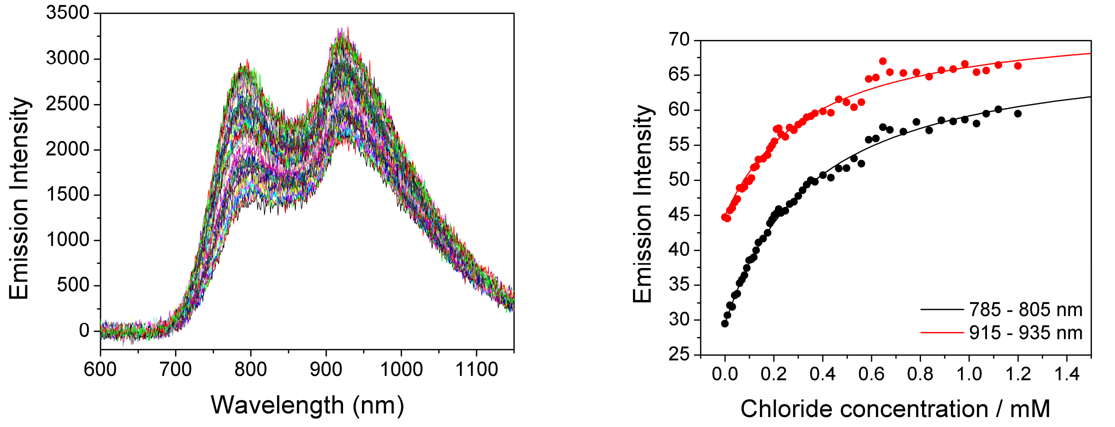

SI 8. Titration of a 4×10^{-5} M solution of macrocycle **4** with TBA.Cl in 97:3 acetonitrile: water. *Left:* showing the changes in the emission spectra upon 430 nm excitation. *Right:* the binding isotherms for Os(bipy)$_3$-based emission bands upon 430 nm excitation: in each case, the line shows the best fit obtained.

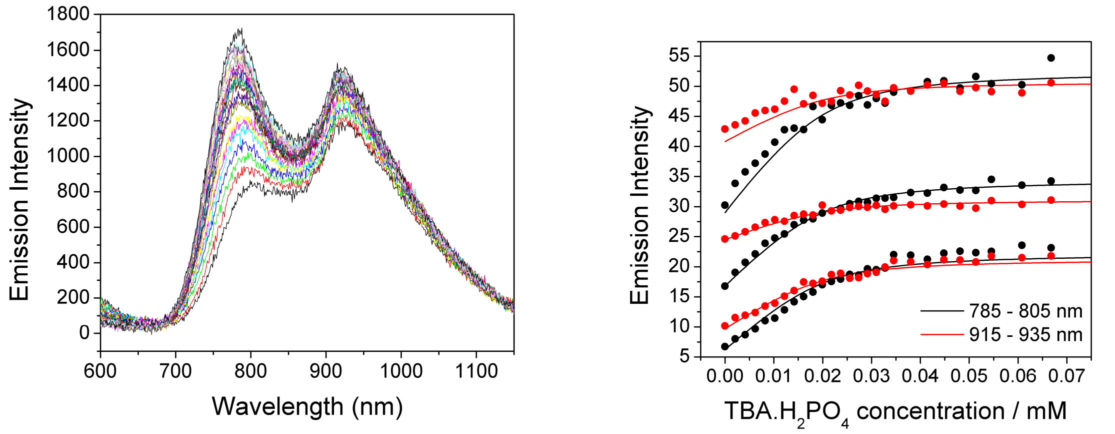

SI 9. Titration of a 2×10^{-5} M solution of rotaxane **1** with TBA.H_2PO_4 in 97:3 acetonitrile: water. *Left:* showing the changes in the emission spectra upon 430 nm excitation for one of the three titrations performed. *Right:* the binding isotherms for Os(bipy)$_3$-based emission bands upon 430 nm excitation for all three titrations: in each case, the line shows the best fit obtained for a global fit of all the data.

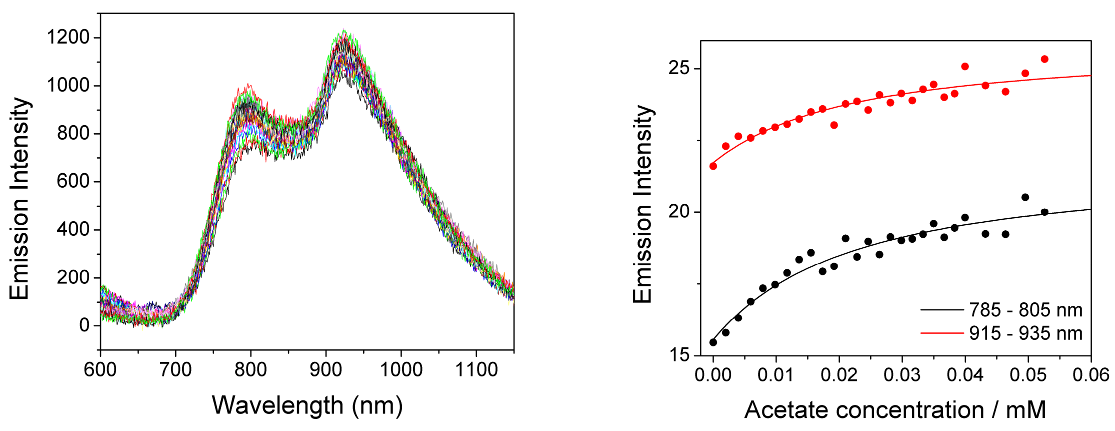

SI 10. Titration of a 2×10^{-5} M solution of rotaxane **1** with TBA.OAc in 97:3 acetonitrile: water. *Left:* showing the changes in the emission spectra upon 430 nm excitation. *Right:* the binding isotherms for Os(bipy)$_3$-based emission bands upon 430 nm excitation: in each case, the line shows the best fit obtained.

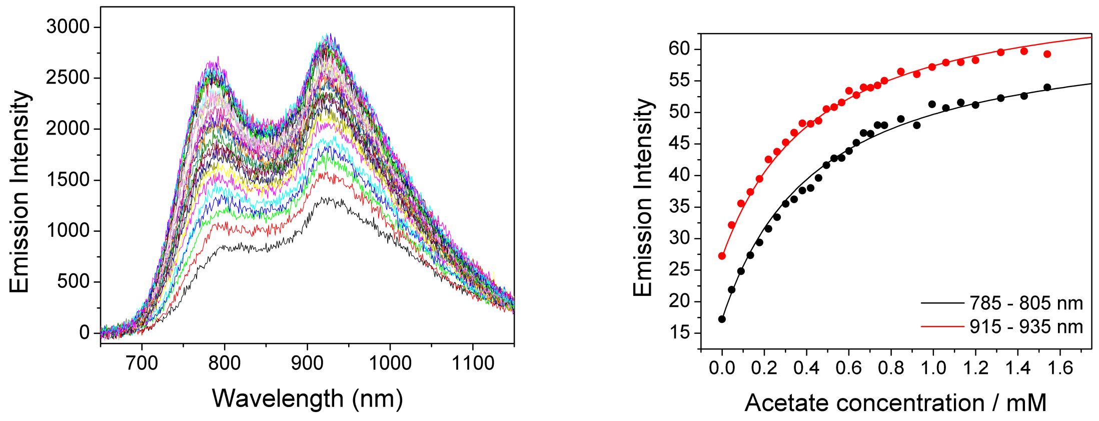

SI 11. Titration of a 4×10^{-5} M solution of macrocycle **4** with TBA.OAc in 97:3 acetonitrile: water. *Left:* showing the changes in the emission spectra upon 430 nm excitation. *Right:* the binding isotherms for Os(bipy)$_3$-based emission bands upon 430 nm excitation: in each case, the line shows the best fit obtained.

Typical Dynafit script (1:1 binding):

```
[task]
data = equilibria
task = fit

[mechanism]
Os + Cl <==> complex1 : K1 assoc

[constants]
K1 = 4E5 ??

[concentrations]
Os = 1.7E-5

[equilibria]
variable Cl

file .\osmium\s1_785_805.txt | response complex1 = 2E9 ? , Os = 1.145E9
file .\osmium\s1_915_935.txt | response complex1 = 2.5E9 ? , Os = 1.745E9

[output]
directory .\output\s1_cl\

[end]
```

References

1. J. Tkac and J. J. Davis, *J. Electroanal. Chem.*, 2008, **621**, 117-120.
